# Supplementary material for: Accuracy of the Lotka-Volterra model fails in strongly coupled microbial consumer-resource systems
Source: PLoS Comput Biol. 2025 Dec 3;21(12):e1013719. doi: 10.1371/journal.pcbi.1013719 (PMC12688139; doi:10.1371/journal.pcbi.1013719)
Supplement: Section A in S1 Appendix — Step-by-step derivation of the GLVA from the MiCRM. Section B in S1 Appendix. Stability analysis. Linearization and stability analysis for the MiCRM. Section C in S1 Appendix. Quantifying separation of timescales. Motivating the importance of timescale separation and deriving an approximate measure of consumer-resource coupling. Section D in S1 Appendix. Balanced sampling of competitive and cooperative communities. Details on the method for balanced sampling of communities with varying degrees of competition and cooperation. (PDF) [file pcbi.1013719.s001.pdf]

Supplementary Materials

A - The Generalised Lotka-Volterra Approximation

Assuming a regime where resources achieve equilibrium much faster than consumers allows us to express the MiCRM dynamical equations solely in terms of the consumers and their interactions. Furthermore, we can reconfigure the terms in a way that gives us an equivalent, "effective" generalized Lotka-Volterra model.

$$\frac{dC_i}{dt} = r_i C_i + C_i \sum_{j=1}^{N} \alpha_{ij} C_j \tag{1}$$

Fast resource equilibration implies that $\frac{dR_\beta}{dt} = 0$, giving us the following relationship:

$$\rho_\beta - \sum_{i=1}^{N} u_{i\beta} R_\beta C_i + \sum_{\alpha=1}^{M} l^i_{\alpha\beta} u_{i\alpha} C_i R_\alpha = 0 \tag{2}$$

Solving for $R_\beta = \hat{R}_\beta$, we can plug it back into the equation for consumers and perform a Taylor expansion of $\hat{R}_\beta$ around the set of equilibrium populations $C_j = \hat{C}_j$.

$$\frac{1}{C_i}\frac{dC_i}{dt} = \sum_{\alpha=1}^{M} (1 - \sum_{\beta=1}^{M} l^i_{\alpha\beta}) u_{i\alpha} \hat{R}_\alpha(\{C_j\}) - m_i \tag{3}$$

Expanding the Taylor series of $\hat{R}_\beta(\{C_j\})$ around equilibrium we have:

$$\hat{R}_\beta(\{C_j\}) = \hat{R}_\beta + \sum_{j=1}^{N} \frac{\partial \hat{R}_\beta}{\partial C_j}(C_j - \hat{C}_j) + \mathcal{O}\left((C_j - \hat{C}_j)^2\right) \tag{4}$$

Our final expression for consumer dynamics is then

$$\frac{1}{C_i}\frac{dC_i}{dt} = \sum_{\alpha=1}^{M} (1 - \sum_{\beta=1}^{M} l^i_{\alpha\beta}) u_{i\alpha} \left(\hat{R}_\alpha + \sum_{j=1}^{N} \frac{\partial \hat{R}_\alpha}{\partial C_j}(C_j - \hat{C}_j) + \mathcal{O}\left((C_j - \hat{C}_j)^2\right) \right) - m_i \tag{5}$$

Here $\frac{\partial \hat{R}_\beta}{\partial C_j}$ can be obtained through direct differentiation of $\frac{dR_\beta}{dt}$, yielding:

$$\left(\sum_{\alpha=1}^{M} \frac{\partial \hat{R}_\alpha}{\partial C_j} \frac{\partial \rho_\beta}{\partial \hat{R}_\alpha} \right) - \sum_{i=1}^{N} u_{i\beta} \left(C_i \frac{\partial \hat{R}_\beta}{\partial C_j} + \delta_{ij} \hat{R}_\beta \right) + \sum_{\alpha=1}^{M} \sum_{i=1}^{N} l^i_{\beta\alpha} u_{i\alpha} \left(C_i \frac{\partial \hat{R}_\alpha}{\partial C_j} + \delta_{ij} \hat{R}_\alpha \right) = 0 \tag{6}$$

Solving the equation for the $\frac{\partial \hat{R}_\beta}{\partial C_j}$ we obtain an expression in the form:

$$\sum_{\alpha=1}^{M} A_{\beta\alpha} \frac{\partial \hat{R}_\alpha}{\partial C_j} = \sum_{\alpha=1}^{M} u_{j\alpha} \hat{R}_\alpha (\delta_{\beta\alpha} - l^j_{\beta\alpha}) \tag{7}$$

where

$$A_{\alpha\beta} = \frac{\partial \rho_\alpha}{\partial \hat{R}_\beta} + \sum_{i=1}^{N} u_{i\beta} \hat{C}_i (l^i_{\alpha\beta} - \delta_{\alpha\beta}) \tag{8}$$

Inverting $A_{\alpha\beta}$ (assuming it is an invertible matrix), we are able to obtain the partial derivatives explicitly.

$$\frac{\partial \hat{R}_\alpha}{\partial C_j} = \sum_{\beta=1}^{M}\sum_{\gamma=1}^{M} A^{-1}_{\alpha\beta} u_{j\beta}(\delta_{\beta\gamma} - l^j_{\beta\gamma})\hat{R}_\gamma \tag{9}$$

Finally, to obtain the effective Lotka-Volterra system, we must group terms and find the equivalent characteristic growth rates r_i, and interaction coefficients α_{ij}. After some algebra, we have the interaction matrix coefficients

$$\alpha_{ij} = \sum_{\alpha=1}^{M} u_{i\alpha}(1 - l^i_\alpha)\frac{\partial \hat{R}_\alpha}{\partial C_j}, \tag{10}$$

and their intrinsic growth rates,

$$\begin{aligned} r_i &= \sum_{\alpha=1}^{M} u_{i\alpha}(1 - l^i_\alpha)\left[\hat{R}_\alpha - \sum_{j=1}^{N}\hat{C}_j\frac{\partial \hat{R}_\alpha}{\partial C_j}\right] - m_i \\ &= \sum_{\alpha=1}^{M} u_{i\alpha}(1 - l^i_\alpha)\hat{R}_\alpha - \sum_{j=1}^{N}\alpha_{ij}\hat{C}_j - m_i \end{aligned} \tag{11}$$

Having defined these coefficients, we can construct an equivalent effective GLV model for any given system of consumers and resources. The prerequisite is that our initial MiCRM system of equations must reach equilibrium asymptotically and that these equilibrium values ($\hat{C}_i$ and $\hat{R}_\alpha$) be known.

We note that the net interaction of one species on another (α_{ij}) is the sum of the partial derivatives of the equilibrium resource concentrations with respect to the abundance of consumer N_j, weighted by the leakage-corrected uptake of consumer N_i. Hence, the sign of α_{ij} predominantly depends on whether these partial derivatives are negative, and to a smaller extent on the weights imposed by uptake and leakage. On average, the interaction coefficients are predominantly negative and the prevalence of weak negative to positive interactions increases with leakage.

B - Stability Analysis

To quantify the characteristic timescales of both consumers and resources, as well as the stability of the feasible (equilibrium) microbial communities, we analyze the behavior of the linearized system about equilibrium.

We begin with trivial equilibrium solutions, $\overline{X}$ and $\overline{Y}$:

$$\begin{aligned} \phi(t) &= X(t) - \overline{X} \\ \Lambda(t) &= Y(t) - \overline{Y} \end{aligned}$$

Next, we reevaluate them at a small perturbation away from the fixed points. For this, we differentiate them, which gives

$$\begin{aligned} \dot{\phi} = \dot{X} = f(X,Y) = f(\overline{X} + \phi, \overline{Y} + \Lambda) \\ \dot{\Lambda} = \dot{Y} = g(X,Y) = g(\overline{X} + \phi, \overline{Y} + \Lambda) \end{aligned}$$

To simplify the analysis, we combine our dependent variables X and Y into a vector Z, and the response functions $f(X,Y)$ and $g(X,Y)$ into a single response function $H(Z) = H(\overline{Z} + \nu)$, with ν our combined perturbation vector. We then perform a Taylor expansion around $\overline{Z}$

$$H(\overline{Z} + \nu) = H(\overline{Z}) + \nu H'(\overline{Z}) + \mathcal{O}(\nu^2) \tag{12}$$

By definition $H(\overline{Z}) = 0$, and since our perturbation is small ($\nu << 1$) we neglect higher-order terms, thus

$$\dot{\nu} = \nu H'(\overline{Z}) \tag{13}$$

Where $H'(\overline{Z})$ is the Jacobian of the response function evaluated at the fixed points $\overline{Z}$. With this linearization, we can evaluate the behavior around equilibrium for any small perturbation ν given that $\nu H'(\overline{Z})$ does not contain any non-trivial zeroes. Additionally, by performing an eigensolution decomposition of our linearized system, we can gain some insight into the stability of any non-hyperbolic fixed point (that is, fixed points for which the real parts of the eigenvalues of the Jacobian are non-zero).

To proceed, we must first explicitly derive the Jacobian of our system. We first note that the set of state variables $\{Z_i\}$ for $i \in \{1, \ldots, N\}$ is equivalent to $\{C_i\}$ and $\{Z_i\} = \{R_\alpha\}$ for $i \in \{N+1, \ldots, N+M\}$. Now, we can derive the diagonal terms for the consumer portion of the Jacobian ($i = \{1, \ldots, N\}$).

$$J_{ii}(\overline{Z}) = \frac{\partial}{\partial C_i} H_i(\overline{Z}) = \sum_{\alpha=1}^{M} (1 - l_\alpha^i) u_{i\alpha} \overline{R}_\alpha - m_i \tag{14}$$

And the off-diagonal terms for the consumer portion are simply zero, $i, j \in \{1, \ldots, N\}$:

$$J_{ij}(\overline{Z}) = 0 \quad \text{for} \quad i \neq j \tag{15}$$

The off-diagonal terms for consumers with respect to resources are $\alpha \in \{N+1, \ldots, N+M\}$ $i \in \{1, \ldots, N\}$:

$$J_{i\alpha}(\overline{Z}) = \overline{C}_i (1 - l_\alpha^i) u_{i\alpha} \tag{16}$$

For the resource states, we can see that the diagonal terms will be $\alpha \in \{N+1, \ldots, N+M\}$:

$$J_{\alpha\alpha}(\overline{Z}) = \frac{\partial}{\partial R_\alpha} H_\alpha(\overline{Z}) = \frac{\partial \rho_\alpha}{\partial R_\alpha} + \sum_{i=1}^{N} u_{i\alpha} \overline{C}_i (l_{\alpha\alpha}^i - 1) \tag{17}$$

The off-diagonal terms with respect to consumers, $\alpha \in \{N+1, \ldots, N+M\}$ $i \in \{1, \ldots, N\}$:

$$J_{\alpha i}(\overline{Z}) = \frac{\partial \rho_\alpha}{\partial C_i} + u_{i\alpha} \overline{R}_\alpha (l_{\alpha\alpha}^i - 1) + \sum_{\beta \neq \alpha}^{M} l_{\beta\alpha}^i u_{i\beta} \overline{R}_\beta \tag{18}$$

And finally, the off-diagonal terms with respect to resources, $\alpha \neq \beta$ $\alpha, \beta \in \{N+1, \ldots, N+M\}$:

$$J_{\alpha\beta}(\overline{Z}) = \frac{\partial \rho_\alpha}{\partial R_\beta} + \sum_{i=1}^{N} l_{\beta\alpha}^i u_{i\beta} \overline{C}_i \tag{19}$$

C - Quantifying separation of timescales (consumer-resource coupling)

To give a better intuition for the relevance of time-scale separation, we illustrate using a simple predator-prey system.

$$\frac{dx}{dt} = \frac{y}{\rho} \tag{20}$$

$$\frac{dy}{dt} = \frac{r}{\rho} y - mx + F(t) \tag{21}$$

Here, predator density (x) is entirely dependent on prey abundance (y), whereas prey naturally reproduce at rate r/ρ, are depleted proportional to predator density (mx), and experience migration betweeen an external population described by the time-varying function $F(t)$. Rearranging terms, we can express this predator-prey system as a second-order differential equation:

$$\rho \frac{d^2x}{dt^2} + mx - r\frac{dx}{dt} = F(t) \tag{22}$$

In this form, the system is entirely analogous to a driven harmonic oscillator with friction. In the absence of migration – $F(t) = 0$ – the solution is simply:

$$x_h(t) = x_0 e^{(-\alpha + i\gamma)t} + x_1 e^{(-\alpha - i\gamma)t} \tag{23}$$

With $\alpha = \frac{r}{2\rho}$ and $\gamma = \sqrt{\frac{m}{\rho} - \alpha^2}$. For an arbitrary $F(t)$, the general solution can be obtained by applying a Laplace transform to both sides of equation 23.

$$\mathcal{L}\left[\rho \frac{d^2x}{dt^2} + mx - r\frac{dx}{dt}\right](s) = \mathcal{L}[F(t)](s)$$

Employing the properties of the Laplace transform and re-arranging terms, we obtain the following relationship, where $\hat{x}(s)$ and $\hat{F}(s)$ denote the Laplace transforms of $x(t)$ and $F(t)$, respectively:

$$\hat{x}(s) = \hat{F}(s)\left[\frac{1}{(s+\alpha)^2 + \gamma^2}\right] + \left[\frac{sx(0) + x(0) - 2\alpha x'(0)}{(s+\alpha)^2 + \gamma^2}\right] \tag{24}$$

Taking the inverse transform of $\hat{x}(s)$, we note that $x(t)$ is simply the solution to the damped harmonic oscillator (eq. 23) in addition to the convolution of $F(t)$ with an exponentially decaying sine wave.

$$x(t) = x_h(t) + \frac{1}{\gamma}\int_0^t F(t-\tau)e^{-i\alpha\tau}\sin(\gamma\tau)d\tau \tag{25}$$

If we take $F(t)$ to represent a coupling force between the oscillator and some external system (e.g. migration or external resource supply), we can ask when $x(t)$ can be treated independently of $F(t)$'s evolution. As is evident from equation 25, this is only possible when the convolution term (which we denote $F_*(t)$) is vanishingly small. If we consider $F(t)$ to be a periodic function or a decaying perturbation, then the question of whether $F_*(t) << x_h(t)$ becomes a matter of relative timescales.

For example, if $F(t) = \cos(\omega t)$, then the convolution becomes

$$F_*(t) = \frac{1}{\gamma}\int_0^t \cos(\omega t - \omega\tau)e^{-i\alpha\tau}\sin(\gamma\tau)d\tau \tag{26}$$

In general, $F_*(t)$ will decay to zero asymptotically and $x(t)$ will converge to $x_h(t)$. However, unless $\omega >> \gamma$, this decay will occur slowly, dominated by α. In other words, the deviation from $x_h(t)$ will persist until equilibrium unless $F(t)$ evolves on a faster timescale. For a decaying perturbation such as $F(t) = a_0 e^{-at}$, the argument is much the same. If $a >> \alpha$, then $F_*(t)$ will quickly approach zero. Otherwise, $F_*(t)$'s convergence will be dominated by α.

Before proceeding, it is important to note that, unlike the unilateral coupling function presented in this short example, consumer-resource coupling is bidirectional. That is, F is a function of $x(t)$ and its derivatives, $F = F(t, x^{(n)})$. This complicates matters significantly as deviations will no longer be bounded and it becomes possible for $x(t)$ to assume a different trajectory.

Having defined the MiCRM's Jacobian, it is possible to estimate the characteristic timescales of consumers and resources. Though this can be done rigorously by identifying the slow and fast manifolds, we present a simplified method that relies on the diagonal elements of the Jacobian. We first note that the decay rate following a small perturbation to either a single consumer or resource is given by:

$$\dot{v}_i = \epsilon \hat{e}_i \mathbf{J} \tag{27}$$

Here $\epsilon << 1$ and $\hat{e}_i$ is the basis vector for the ith consumer or resource. It can be shown that return times following such a perturbation are inversely proportional to the corresponding diagonal element of the Jacobian.

$$\tau_i = \frac{1}{J_{ii}} \tag{28}$$

Hence, for consumers, we have

$$\tau_{Ci} = \left[\sum_{\alpha=1}^{M}(1 - l^i_\alpha)u_{i\alpha}\overline{R}_\alpha - m_i\right]^{-1} \tag{29}$$

And for resources

$$\tau_{R\alpha} = \left[\frac{\partial \rho_\alpha}{\partial R_\alpha} + \sum_{i=1}^{N} u_{i\alpha}\overline{C}_i(l^i_{\alpha\alpha} - 1)\right]^{-1} \tag{30}$$

This is guaranteed as long as the Jacobian is diagonally dominant, which is typically true in the MiCRM. Even when this condition is not satisfied, it is easy to see that the diagonal elements of the Jacobian are generally much larger, hence we expect this to be a reasonable approximation. Next, we assume that consumer-resource coupling across the community is dominated by the fastest consumer and the slowest resource. We justify this assumption by noting that the presence of a single consumer-resource pair progressing at similar temporal scales can have cascading effects on the rest of the community; making it a sufficient condition for the violation of the fast resource dynamics assumption.

As such, finding the timescale ratio between consumers and resources is equivalent to:

$$\varepsilon = \min_{\forall i, \forall \alpha}\left(\frac{\tau_{Ci}}{\tau_{R\alpha}}\right) \tag{31}$$

This is analogous to finding

$$\tau_C = \min_{\forall i}(\tau_{Ci}) \tag{32}$$

$$\tau_R = \max_{\forall \alpha}(\tau_{R\alpha}) \tag{33}$$

$$\tag{34}$$

Hence:

$$\varepsilon = \frac{\tau_C}{\tau_R} \tag{35}$$

D - Balanced sampling of competitive and cooperative communities

To ensure that the community sampling procedure provides a well-represented range of competitive and cooperative structures, we designed the following method to vary parameters such that a desired average niche and effective leakage overlap is attained. We begin by constructing the consumer preference matrix. To this end, we define two extremes of the sampling procedure exemplified by a zero overlap matrix θ_{id}, which is simply an identity matrix, and the maximum overlap matrix θ_{hom}, which is a matrix populated by ones. The idea here is that we can define our overall consumer preference matrix as the weighted sum of the zero- and maximum-overlapping matrices; varying the weights in each extreme allows us to continuously move from zero to maximum overlap.

$$\theta = w_{id}\theta_{id} + w_{hom}\theta_{hom}$$

Having defined a method to continuously move between both edges of the overlap of consumer preferences, we now do the same with our specificity parameter Ω. The motivation behind this is that the specificity must be high if we want to reliably sample communities with negligible overlap. Conversely, as consumer preferences become more similar, we want specificity to be lower, allowing

for greater variability and avoiding drawing the same highly homogeneous consumer uptake matrix multiple times.

$$\Omega = W_{id}\Omega_{id} + W_{hom}\Omega_{hom}$$

With this framework, we can define a niche similarity parameter s_n such that $w_{id}(s_n)$ and $w_{hom}(s_n)$ are decreasing and increasing functions of s_n respectively. Applying a similar argument to specificity, we need $W_{id}(s_n)$ to be an increasing function and $W_{hom}(s_n)$ to be decreasing. In short, we need the weights to be inversely proportional with respect to niche similarity, in this manner varying s_n between some arbitrary interval (say [0-1]), produces communities that go from no niche overlap to maximum niche overlap.

The stoichiometric matrix is sampled similarly to consumer uptake. However, the goal in this context is to create communities that exhibit a diverse array of cross-feeding behaviors. This is further complicated by the fact that cross-feeding is largely dependent on consumer preferences. To circumvent this problem, we sampled consumer leakage based on the maximum and minimum amounts of cross-feeding achievable for a given set of consumer preferences.
