# Supplementary material for: Accuracy of the Lotka-Volterra model fails in strongly coupled microbial consumer-resource systems
Source: PLoS Comput Biol. 2025 Dec 3;21(12):e1013719. doi: 10.1371/journal.pcbi.1013719 (PMC12688139; doi:10.1371/journal.pcbi.1013719)
Supplement: Attachment — Submitted filename: Response_to_Reviewers.pdf [file pcbi.1013719.s002.pdf]

Imperial College
London

Department of Life Sciences
Imperial College London (Silwood Park Campus)
Buckhurst Road, Ascot
Berkshire SL5 7PY, UK

23 September 2025

Dear Editor,

We thank the Reviewers for their detailed reading of our manuscript, constructive comments, and overall positive endorsement of its findings.

We have revised the manuscript to address the concerns of all Reviewers.

The following is a list of the main issues raised by the reviewers, and our revisions to address each of them:

- **Biological realism and experimental connection.** Reviewers asked whether predicted behaviors (e.g. overshoot, stability differences) occur in real microbial systems and questioned the biological plausibility of leakage values. *In response*, we have pointed out that the carrying capacity overshoot is neither general nor exclusive to the MiCRM, and added empirical references justifying the leakage range we consider in the methods section.
- **Scope of comparison (analytical vs. inferential GLV).** Reviewers noted that the manuscript treats the MiCRM as "ground truth" and compares it only to the analytical GLVA, not to statistically inferred GLVs. *In response*, we clarified our focus on the analytical GLVA as a first-order representation of the MiCRM, and explained our choice of focus in the context of inference-based studies (with references).
- **Model assumptions (QSS, additivity, linearisation).** Reviewers questioned the validity of the quasi–steady-state assumption, the choice of linearising around consumer equilibrium, and the additivity of consumer growth across resources. *In response*, we expanded the Discussion to further clarify the QSS condition in terms of timescale separation and biomass partitioning, have noted alternative linearisation choices are possible, and added text justifying our focus on systems where growth is additive with respect to multiple resources.
- **Interpretation of GLVA failures and interaction coefficients.** Reviewers asked whether GLVA's failures are due to higher-order interactions or misspecified pairwise interactions, and sought clarification on how interaction coefficients are determined. *In response*, we have added an explanation that deviations necessarily arise from higher-order terms in the analytical approximation, and clarified the interpretation of α_{ij}, noting the predominance of competitive interactions with facilitation increasing under high leakage.
- **Metrics of accuracy, stability gap, and discussion of limitations.** Reviewers criticised the use of categorical accuracy metrics, requested a deeper explanation of stability

> differences, and asked for a stronger discussion of limitations and future directions. *In response*, we replaced categorical thresholds with continuous error measures (completely revised Fig. 2 & 4), expanded our interpretation of the stability gap, and added explicit discussion of limitations and potential future extensions.

Our point-by-point responses are attached.

Yours sincerely,

Michael Mustri, Quqiming Duan, and Samraat Pawar

Responses to the Reviewers

Comments made by Reviewers are in **blue text**, whereas our responses are in **red text**.

All revisions to the manuscript referenced below are in **red text** in the revised manuscript.

Reviewer #1

1. What about contrasting either GLV or MiCRM equations against experimental data? As shown in Fig. 1, the MiCRM generally produces an initial overshooting of population abundances that is absent in the GLVA. A significant portion of the trajectory error arises from this discrepancy compared to the GLVA. Is such initial overshooting actually observed in experiments with microbial communities?

   This is a good point, and we appreciate it being raised, as it draws attention to the need for high-quality, multispecies microbial growth curves. It is challenging to experimentally measure population growth trajectories with the necessary resolution in multi-species communities. Despite this, population overshoot is a well-documented phenomenon in microbial communities (Vadasz et al., 2001; Caperon, 1969), typically attributed to a delayed response to changing environmental conditions. In the MiCRM, this arises "naturally" as growth rate starts high and tapers off as resources become depleted. Of course, the example of Fig. 1 (which we have now changed) is not generally representative of the diversity of dynamical behaviors achievable in either model. The GLVA is capable of reproducing this overshoot in some instances, and there are times when the overshoot is not present in the MiCRM.

2. In Fig. 3, the color codes for some of the leakage values are almost invisible. Please correct this.

   Fixed. Changed color palette to make contrast stronger and added a colour bar to avoid readability issues.

3. L. 372: Regarding the importance of higher-order interactions to overcome the GLVA failures: A model's failure might actually stem from a misspecified pairwise interaction, rather than the presence of higher-order interactions, as pointed out by Case and Bender (1981).

   While this is true, it is unlikely that the GLVA's failure is a result of misspecified interactions. To illustrate why this is the case, we note that all the examples discussed by Case and Bender Case and Bender (1981) pertain to statistically inferred interactions from empirical systems. Given that the GLVA is an analytical approximation to the MiCRM, the calculated interaction coefficients are an exact representation of first-order terms. As

such, any deviations observed in the approximation must necessarily arise due to the presence of higher-order terms. These deviations could, of course, be eliminated by including second and third order terms, but that would defeat the purpose of the approximation as it would exceed the complexity of the MiCRM itself. We have added a few sentences to justify our method at the end of the introduction (lines 96-112)

4. Appendix A: A question about Eq. (15), which defines the interaction matrix coefficients a_{ij} as a sum of products. Specifically, of $u_{i\alpha}$ times the proportion of resources leaked times the partial derivative of R_a respect to C_j. Given that the first two factors are, by definition, non-negative, it appears that the sign of α_{ij} is primarily determined by the sign of the partial derivative $\frac{\partial C_j}{\partial R_\alpha}$. Is this interpretation correct? How do you interpret this dependency? Do you have an estimate of the relative prevalence or weights of competitive (negative) versus facilitative (positive) interactions predicted by this formulation?

   This is indeed an accurate interpretation. To expand on it, we note that the net interaction of one species on another (α_{ij}) is the sum of the partial derivatives of the equilibrium resource concentrations with respect to the abundance of consumer N_j, weighted, of course, by the leakage-corrected uptake of consumer N_i. Hence, the sign of α_{ij} predominantly depends on whether these partial derivatives are negative, and to a smaller extent on the weights imposed by uptake and leakage. We do not have a precise estimate, but the interaction coefficients are predominantly negative, though the prevalence of weak negative to positive interactions increases with leakage. We have now clarified this in the revised Appendix A.

5. L. 105: MacArthur appears twice.

   Fixed

6. In the equation of the error trajectory, above L. 183, a time differential, dt, is lacking in the integral.

   Fixed

Reviewer #2

Major Comments:

7. Throughout, implicitly the MiCRM was taken to be "truth" and the GLVA was compared to this truth. It should be noted that the GLVA as derived is not the same as what would be inferred by using a statistical method to fit the time series data generated by a MiCRM. For example, the best fit GLV model to the MiCRM time series in figure 1 might look far more like the MiCRM time series. Also, it would have different stability

and reactivity than the GLVA. It is not at all clear from the analyses in this manuscript that an inferred GLV model would actually differ in (for example) estimated stability and reactivity in the same way the GLVA differs.

This is an excellent point and we acknowledge that it is generally true. In this paper we have limited ourselves to a comparison between the "true" MiCRM and it's first order Lotka-Volterra approximation for two main reasons. First, there are many other research papers which have investigated the behaviour of statistically inferred Lotka-Volterra models relative to the corresponding dynamics of substrate mediated interactions (see for example Letten and Stouffer (2019); Momeni et al. (2017); Dedrick et al. (2023)). The second reason is that, by assessing the performance of an exact first order representation of resource mediated interactions (that is, the GLVA) we can gain more insight into the theoretical limitations of pairwise frameworks as opposed to the methodological challenges. We have added a few sentences to justify our method at the end of the introduction **We have addressed this issue in lines 96-112 of the manuscript.**

8. Throughout, error was defined by how often and by how much the population dynamics deviate rather than a comparison of the nature and relative strengths of the interactions themselves. Often the goal of fitting a GLV model to data is not to make predictions about future dynamics, but rather to study the interactions themselves. Even if the dynamics differ somewhat, are the nature of interactions accurately estimated (for example negative interactions from resource competition or positive interactions from cross feeding)? To me, this would be a more interesting test of the accuracy of the GLV even if dynamics differ subtly.

   We are aware of the limitations involved in out method for quantifying error rate, however, we will specifically address the challenge of comparing interactions between models. Given the fundamental differences between the MiCRM and the GLV (namely, that they differ in dimensionality), there is no straightforward way by which interactions in both models may be directly compared. One method would be to perform a perturbation analysis for each pair of consumers and numerically estimate the interaction coefficients as the corresponding deviations from equilibrium (see, for example, Cook Id et al. (2021)). However, aside from the computational challenge implicit in this task, it is worth noting that the GLVA analytically captures this first-order effect, and we would expect almost exact first-order agreement between the numerical and analytical solutions.

9. The GLV approximation is performed by taking the resources to quasi steady state and then linearizing the per capita rates about the equilibrium. Mathematically, the QSS assumption is identical to setting dR/dt=0 as stated in the text in the Appendix. But what is actually being assumed is not that R is at equilibrium and not changing, but rather that it changes very rapidly so that there is a small parameter epsilon such that epsilon*dR/dt=f(R,C), and then epsilon is set to zero. Intuitively, I believe that this

requires that nearly all biomass is tied up in consumers C rather than as free biomass R (large uptake rates relative to the decay rates m and relatively low leakage). Essentially, free resource would be nearly instantly taken up by consumers if the QSS assumption is valid. It seems to me that leakage increases the stability gap because it makes the QSS assumption invalid (resources would no longer be fast relative to consumers). Working this through formally may show exactly which combinations of parameters need to be small for the approximation to be good. The proportion of free resource at steady state would also be measurable, so if my intuition above is correct it would be something that could be ascertained relatively easily in real systems.

Yes, we agree wholeheartedly with this interpretation and point out that it is consistent with our results, particularly in reference to the timescale analysis. Looking closer at equations 34 and 35 in Appendix C, we can note a few key parameters that lead to the failure of the QSS assumption (which we have referred to as the timescale separation assumption). First, higher leakage leads to slower resource timescales, and the opposite is true of consumers. However, and more to your point, if a larger proportion of the biomass is in consumers at equilibrium this implies $\sum \hat{C}_i >> \sum \hat{R}_\alpha$, which would indeed lead to a more robust separation of timescales as seen from equations 32 and 33. The characteristic timescales capture this relationship more rigorously and comprehensively, but acknowledging empirical limitations, we have included this perspective in the discussion (lines 425-429)

10. I don't think that the linearization for the approximation of the GLV line 381-384 need not be around the equilibria for C in order to create a GLV model. This is of course a natural choice because it ensures that the GLV will have the same equilibria as the consumer resource model, but we wouldn't expect the approximation to work well as C deviates from its equilibrium. Other choices might make sense in other scenarios, such as wanting to predict the initial growth rates of consumers when given a suite of resources (community assembly).

    This is a good observation and we agree that linearizing at the non-trivial equilibrium is a somewhat arbitrary choice. The linearization procedure would be identical if performed around any hyperbolic fixed point. Additionally, the system could be linearized at the exponential growth phase, though this implies a rearrangement of the differential equations. Of course, we expect the results to hold regardless of which fixed point we choose.

11. The MCRM also makes the assumption that growth rates of consumers due to different resources are additive (just like interactions in the GLV). Namely, if a consumer grows at rate r1 on resource 1 and rate r2 on resource 2, the MCRM model assumes that the growth rate of the consumer will be r1+r2. Yet we know this generally not the case. The consumer may still just grow at rate r1 even when resource 2 is present because it

prefers growth on resource 1 over on resource 2.

While it is true that growth rate does not always scale additively in this regard, the only scenario which considerably changes the dynamics is that of limiting nutrients or non-substitutable resources. Non-substitutable resources can be accommodated into the MiCRM by considering different models of co-limitation (for example, see Cui et al. (2024)). However, there are two main reasons why we did not consider non-additive effects. The first is that it is known that multiplicative co-limitation requires higher order interactions to be effectively reduced to a pairwise model with only consumers (O'Dwyer, 2018), therefore we do not expect the results to meaningfully differ with the addition of leakage. The second reason is that the approximation would require a substantially different approach and the increase in model complexity does not seem warranted given the (relatively) well known higher order dynamics of non-substitutable resources.

12. Line 189: Why were the results classified as accurate, overshot and undershot rather than simply showing the relative error directly? Fig 2: I'd have rather seen a plot of relative error as a function of leakage. Also, I would not consider deviations in predicted dynamics of 20% as large. If we could actually predict even the order of magnitude of dynamics in real systems we would probably be happy.

    We agree that this was a rather arbitrary choice and have modified the results so that they no longer depend on set thresholds for accuracy.

13. How do the chosen leakage levels correspond to real systems? Low leakage is defined as 0.3 but how is this number selected? Why not 0.1 or 0.01? If leakage is typically low in real systems, maybe the GLV provides an adequate approximation. More generally, the results depend heavily on the specific numbers chosen and there is little justification for these from real systems.

    We thank the reviewer for highlighting this important issue. We have now added empirical references and a new paragraph in the Methods justifying our simulated leakage values based on real systems. While leakage rates vary widely across microbes and metabolites, empirical studies report ranges from 1% to >60% depending on context (see papers cited - lines 145–150 of the main text). Thus, our "low" ($l^i_\alpha < 0.3$) to intermediate ($0.3 \leq l^i_\alpha \leq 0.6$) leakage regimes fall within empirically observed ranges, while our high leakage regimes explore a limiting case. We now explicitly state this in the manuscript (lines 176-181).

Minor Comments:

- Figure 1: The vertical axes in both plots should be the same for ease comparison (alternatively these could be shown on the same plot).

Fixed. Increased figure resolution and changed the simulation parameters to show more interesting comparison.

- Figure 3: It is very difficult to see the definition of the colors.

  Fixed.

Reviewer #3

Major Comments:

14. Line 207: While the difference in predicted stability is shown clearly, there is not enough discussion on the reason behind this difference in my opinion. I would suggest including the reason (or your speculation) about this difference in stability between the two models.

    We have expanded on our interpretation of the stability results, pointing out some of the likely explanations (lines 350–355).

Minor Comments:

- Line 193: I suggest using a more explicit and direct title for this result section. Using ‘community structure’ is a little vague. Perhaps mentioning niche overlap would be a more direct representation of the concepts discussed in this section

  Changed title to reflect the core contents of the results.

- Figure 3: Please include more information about each panel in the caption to make the figure more accessible.

  We have expanded the description in the caption and pointed out the which panel corresponds to each metric.

- Figure 3: Please make the colors in the legend more prominent (larger dots?)

  Fixed

- In Appendix C, the discussion about the harmonic oscillator is distracting, in my opinion. I think it would be better to focus on the consumer-resource model instead. If an example is needed, I would suggest discussing a simple two-species community in the consumer-resource model for more insight.

  We have made a slight modification to the example to frame it within the familiar context of a predator-prey interaction. We have kept the general formulation the same since it gives a good intuition for the issue of timescale separation without being mathematically intractable.

- Line 224: The title of this section is a little vague. Please consider replacing it with a more direct and explicit title that describes your findings.

  Changed section title to something that better reflects the findings

- The discussion section does a good job of summarizing the findings and linking them to the existing literature, but it's relatively weak in discussing limitations and future directions.

  We have now added text that addresses this comment (lines 430–436).

References

Caperon, J. 1969. Time Lag in Population Growth Response of Isochrysis Galbana to a Variable Nitrate Environment. Ecology 50:188–192. ISSN 1939-9170. URL https://onlinelibrary.wiley.com/doi/abs/10.2307/1934845, _eprint: https://esajournals.onlinelibrary.wiley.com/doi/pdf/10.2307/1934845.

Case, T. J. and Bender, E. A. 1981. Testing for Higher Order Interactions. The American Naturalist 118:920–929. ISSN 0003-0147. URL https://www.jstor.org/stable/2460826, publisher: [The University of Chicago Press, The American Society of Naturalists].

Cook Id, J., Pawar, S., 3, I. D., and Endres Id, R. G. 2021. Thermodynamic constraints on the assembly and diversity of microbial ecosystems are different near to and far from equilibrium URL https://doi.org/10.1371/journal.pcbi.1009643, iSBN: 1111111111.

Cui, W., Marsland, R., and Mehta, P. 2024. Les Houches Lectures on Community Ecology: From Niche Theory to Statistical Mechanics. ArXiv page Arxiv:2403.05497v1. ISSN 2331-8422. URL https://www.ncbi.nlm.nih.gov/pmc/articles/PMC10942479/.

Dedrick, S., Warrier, V., Lemon, K. P., and Momeni, B. 2023. When does a Lotka-Volterra model represent microbial interactions? Insights from in vitro nasal bacterial communities. mSystems 8:e00757–22. URL https://journals.asm.org/doi/full/10.1128/msystems.00757-22, publisher: American Society for Microbiology.

Letten, A. D. and Stouffer, D. B. 2019. The mechanistic basis for higher-order interactions and non-additivity in competitive communities .

Momeni, B., Xie, L., and Shou, W. 2017. Lotka-Volterra pairwise modeling fails to capture diverse pairwise microbial interactions. eLife 6. ISSN 2050084X. Publisher: eLife Sciences Publications Ltd.

O'Dwyer, J. P. 2018. Whence Lotka-Volterra? Theoretical Ecology 11:441–452. ISSN 1874-1746. URL https://doi.org/10.1007/s12080-018-0377-0.

Vadasz, A. S., Vadasz, P., Abashar, M. E., and Gupthar, A. S. 2001. Recovery of an oscillatory mode of batch yeast growth in water for a pure culture. International Journal of Food Microbiology 71:219–234. ISSN 0168-1605. URL https://www.sciencedirect.com/science/article/pii/S0168160501006183.
